# Single-cell dispensing and ‘real-time’ cell classification using convolutional neural networks for higher efficiency in single-cell cloning

**DOI:** 10.1038/s41598-020-57900-3

**Published:** 2020-01-27

**Authors:** Julian Riba, Jonas Schoendube, Stefan Zimmermann, Peter Koltay, Roland Zengerle

**Affiliations:** 1 Cytena GmbH, Neuer Messplatz 3, 79108 Freiburg, Germany; 2 Laboratory for MEMS Applications, IMTEK - Department of Microsystems Engineering, University of Freiburg, Georges-Köhler-Allee 103, 79110 Freiburg, Germany; 3 Hahn-Schickard, Georges-Koehler-Allee 103, Freiburg, 79110 Germany; 4 BIOSS Centre for Biological Signalling Studies, University of Freiburg, Schänzlestr. 18, 79104 Freiburg, Germany

**Keywords:** Recombinant protein therapy, Machine learning

## Abstract

Single-cell dispensing for automated cell isolation of individual cells has gained increased attention in the biopharmaceutical industry, mainly for production of clonal cell lines. Here, machine learning for classification of cell images is applied for ‘real-time’ cell viability sorting on a single-cell printer. We show that an extremely shallow convolutional neural network (CNN) for classification of low-complexity cell images outperforms more complex architectures. Datasets with hundreds of cell images from four different samples were used for training and validation of the CNNs. The clone recovery, i.e. the fraction of single-cells that grow to clonal colonies, is predicted to increase for all the samples investigated. Finally, a trained CNN was deployed on a c.sight single-cell printer for ‘real-time’ sorting of a CHO-K1 cells. On a sample with artificially damaged cells the clone recovery could be increased from 27% to 73%, thereby resulting in a significantly faster and more efficient cloning. Depending on the classification threshold, the frequency at which viable cells are dispensed could be increased by up to 65%. This technology for image-based cell sorting is highly versatile and can be expected to enable cell sorting by computer vision with respect to different criteria in the future.

## Introduction

Up to now, more than 50 monoclonal antibodies for therapeutics have been introduced - most of them for cancer therapies - and the market for drugs based on monoclonal antibodies reached almost 100 billion USD a year^1^. The most widely adopted cell lines for production of these recombinant proteins are engineered Chinese hamster ovarian (CHO) cells. Based on early regulatory guidelines released by the US federal drug administration (FDA) and other regulatory bodies, the production cell line for a recombinant product is to be cloned from a single progenitor cell^2^. This is required in order to minimize population heterogeneity and facilitate isolation and subsequent selection of high producing clones, which could be otherwise overgrown by fast growing but low producing clones. Therefore single-cell cloning is an important step for biopharmaceutical production of monoclonal antibodies.

Several methods for automated single-cell isolation for clonal cell line development have been proposed. The most commonly used methods include limiting dilution^3^, fluorescent-activated cell sorting (FACS)^4^, and single-cell dispensing^5^. Besides the assurance of monoclonality another important aspect of any cloning workflow is to achieve high cloning efficiency, i.e. to maximize the percentage of viable clonal cultures per plate. Using CHO cells and other cell lines, high cell viability and cloning efficiency has been achieved with single-cell dispensing^5,6^. However, in practice, cell samples often contain significant fractions of cells that are dead or are difficult to grow, which can result in low clone recovery. It is not uncommon in industrial cell line development workflows, that only 20% of the isolated single cells grow to usable colonies^7^. This problem might be due to cell damage caused by the sample preparation process. Therefore, a single-cell isolation method, that allows selecting for cells that are likely to proliferate and form a colony, would be of great benefit.

FACS can be used to gate for viable cells based on forward (FSC) and side scatter (SSC) or on fluorescent viability dyes such as propidium iodide or 7AAD, which are DNA intercalating substances that do not pass through intact cell membranes and therefore only label dead cells, or CMFDA or Calcein AM which are membrane-permeable substances that are turned over to fluorescent products by viable cells. However, finding the optimal gating strategy is a manual process which must be repeated for each sample. Further, staining of cells can have an impact on their viability or proliferation.

In contrast to FACS or limiting dilution, in single-cell dispensing the selection of individual cells is based on the assessment of imaging data^6^. A liquid dispenser generates droplets of 50–200 pl in volume on-demand. The nozzle of the dispenser is monitored by a microscopic camera system. Based on the analysis of these images an algorithm decides whether the volume of the next droplet is expected to contain a single cell and to dispense the droplet onto the target (e.g. a microwell plate) or to remove it, for example by a vacuum suction mechanism. Compared to continuous droplet generation utilized in FACS, drop-on-demand dispensing provides flexibility for the timing of the decision-making algorithms. Instead of synchronizing the algorithm with a fixed dispensing frequency, the droplet generation is continuously adapted to the timing of the image processing step.

Previous studies have demonstrated that supervised machine learning can be used to analyze large datasets of single-cell images obtained from high-throughput microscopy^8^ or for classification of images that were obtained by imaging flow cytometry^9^. Very recently, convolutional neural networks (CNNs) have been applied for cell sorting on an imaging flow cytometry setup^10^.

In this work, single-cell dispensing is combined with machine learning in order to increase number of viable clones per microplate. Therefore, the object detection algorithm that identifies single-cells based on size and roundness was extended with an additional classifier that uses CNNs to predict whether a cell is likely to grow. For the first time, drop-on-demand dispensing is combined with machine-learning based image classification for droplet sorting. Here, the term ‘real-time’ used to highlight that cell selection and sorting on the instrument is based on the outcome of the image classification.

## Results and Discussion

### Implementation and training of the classifier for viability prediction

**Figure 1:**
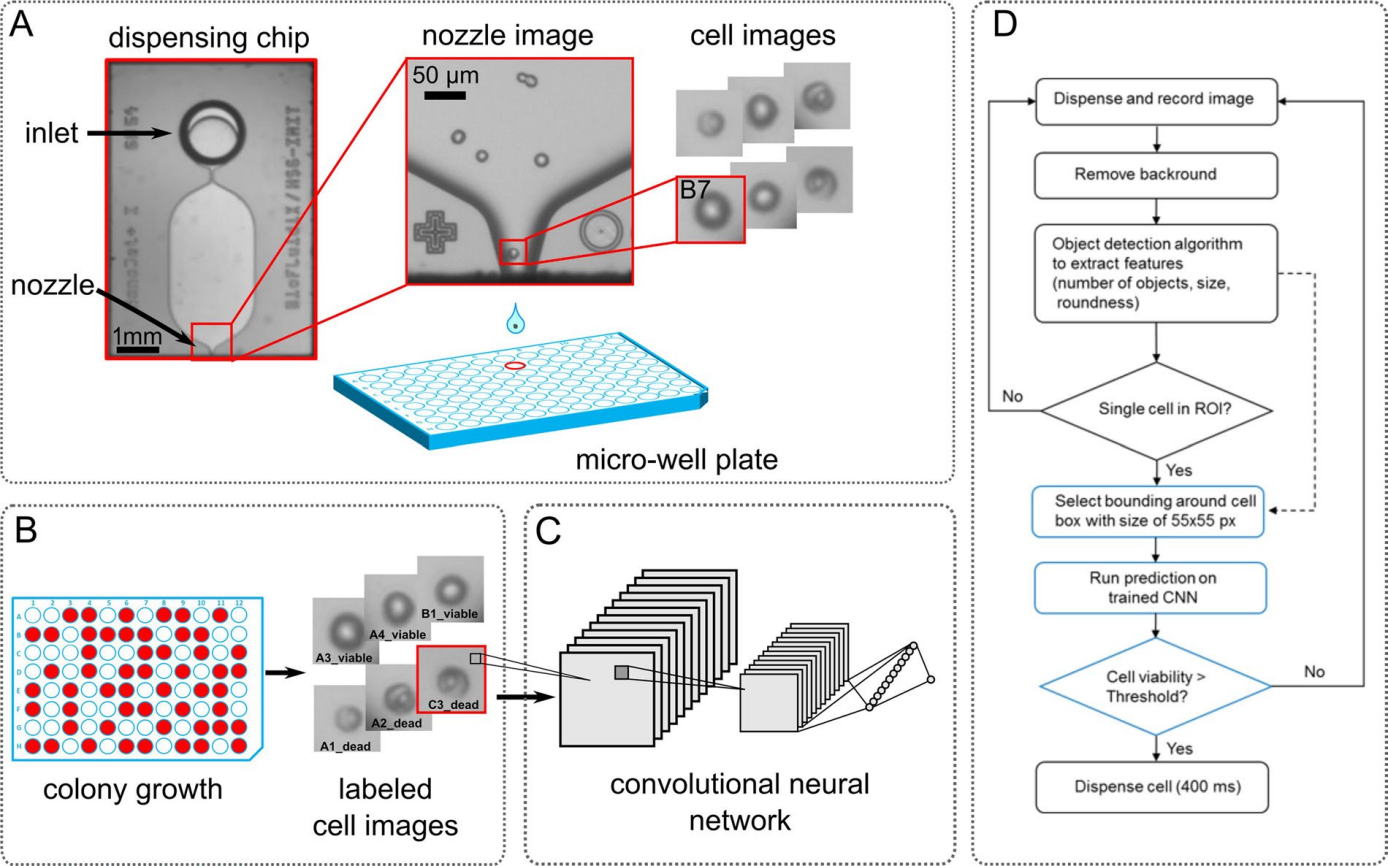
Overview of the training procedure and implementation of the CNN-based classifier. (**A)** Cell isolation: A microscopy image of the silicon/glass dispensing chip is shown. The cell suspension enters the chip via the inlet and ~160 pl droplets are ejected from the 40 µm x 40 µm sized nozzle. Images of the nozzle region are captured by the camera of the cell detection microscope in the single-cell printer. A cropped image of each dispensed single-cell with a size of 55 × 55 pixels is stored and can be unambiguously linked to the microwell the cell was deposited into. **(B)** Cultivation: Colony growth is assessed by imaging the plates. Cells that resulted in viable colonies after 10 days are labeled “viable”, cells that did not grow are labeled “dead”. **(C)** Training: Labeled cell images are used to train a convolutional neural network (CNN) with one output node for binary classification of input images 55 × 55 pixels in size. The flowchart in **(D)** illustrates the implementation of the additional CNN-based cell classifier into the cell detection algorithm of the single-cell printer: Only if the feature-based object detection algorithm identified a single cell in the nozzle, a cropped region with the cell is selected for additional prediction on the trained CNN (indicated in blue).

As shown previously^3,6,11^ the single-cell printer captures images of the region in proximity of the nozzle of a silicon/glass dispensing chip displayed also in Fig. 1A. Both the drop-on-demand dispenser and the highly magnifying vision system are coupled and controlled by a computer which allows for automated detection of single cells inside the dispensing chip. The pneumatic shutter system removes empty and otherwise unwanted droplets. Only droplets that are classified for deposition by the image processing algorithm can pass to the target substrate (e.g. a microwell). The CNN-based classification for viability prediction was implemented into the existing software for cell detection and controlling of the instrument (c.sight single-cell printer, cytena GmbH, Germany) without any hardware changes.

An overview of the training procedure is shown in Fig. 1A–C. During the single-cell isolation images of each individual cell that was dispensed are automatically stored to the hard drive. Here, cropped regions of the image, 55 × 55 pixels in size, centered around the cell, are stored in addition (Fig. 1A). For a posteriori background removal, ‘empty’ images that were captured prior cell dispensing are stored as well. All images can be unambiguously linked to the microwell that was addressed by the dispenser. After single-cell deposition into microwells prefilled with growth medium, the plates are incubated for 10 days before colony growth was assessed by microscopy. Cells that resulted in viable colonies are labeled “viable” while cells that did not grow to visible colonies are labeled “dead” (Fig. 1B). The labeled cell images were then used to train a binary classifier based on a convolutional neural network (CNN) (Fig. 1C). The trained classifier can now be used for ‘real-time’ viability prediction during cell dispensing. Here, the classifier was implemented in addition to the previously described feature-based object detection algorithm^6^, which is based on number of objects, object size and roundness. When this conventional object detection algorithm identifies a single cell in the region of interest (ROI) inside the nozzle, a cropped region of the image with the cell in the center is selected for additional classification by the trained CNN as illustrated in the flowchart in Fig. 1D.

### The concept of a binary classifier for viable cell selection

As outlined above, a binary classifier was trained to select for cells that have a high probability to grow to viable clonal colonies. In the following, several network architectures were trained with different datasets. The performance of a binary classifier on a set of data (the ‘test set’) for which the true values are known, can be expressed as confusion matrix:

To compare the performance of different models on different datasets the following metrics were used:

The accuracy for predicting viable cells correctly is given as true positive rate:

$$TPR=\frac{TP}{{\rm{TP}}+{\rm{FN}}},$$

and the accuracy for predicting dead cells correctly is given as false positive rate:

$$FPR=\frac{TN}{{\rm{TN}}+{\rm{FP}}}.$$

The overall accuracy of a classification model on all cell images is defined as:

$$ACC=\frac{TP+TN}{{\rm{TN}}+{\rm{FP}}+{\rm{FN}}+{\rm{TP}}},$$

where TP, TN, FP and FN stand for the total number of such events according to the confusion matrix. The number of viable colonies that are predicted to grow using the classifier can be derived by multiplying the fraction of ‘viable cells’ in the sample c_v_ with TPR. The total number of dispensed single-cells passing the classifier yields ${c}_{v}TP{R}_{}+\,(1\,-\,{c}_{v})(1-\,FP{R}_{})$, as it also includes the dead cells (1 - c_v_) which incorrectly classified as ‘viable’. In addition to this commonly used metrics, the growth increase GI is defined as the ratio of the number of viable colonies that are predicted to grow using the classifier divided by the number of viable colonies c_v_ that were obtained in experiments not using the classifier.

$${\rm{GI}}=\frac{{c}_{v}TPR}{{c}_{v}TPR+(1-{c}_{v})(1-FPR)}\cdot \frac{1}{{c}_{v}}$$

Most classifiers, such as the ones used here, output a probability P value rather than an actual class label. Here, this probability value ranges between 0 (’dead’) and 1 (’viable’).

The metrics defined above depend on the threshold value $T\,$that is used for classification (for $P > T$ the cell is classified as ‘viable’). The classification performance for different values of $T$ is considered by another metric for binary classification, the area under curve (AUC) which can be retrieved from a receiver operator characteristic (ROC) that plots the true positive rate TPR against the false positive rate FPR for all valid threshold values $T\in \,[0,1]$. An AUC of 1.0 represents a perfect classifier, while a classifier with an AUC of 0.5 is not performing better than random guessing.

### A shallow convolutional neural network for cell classification

**Figure 2:**
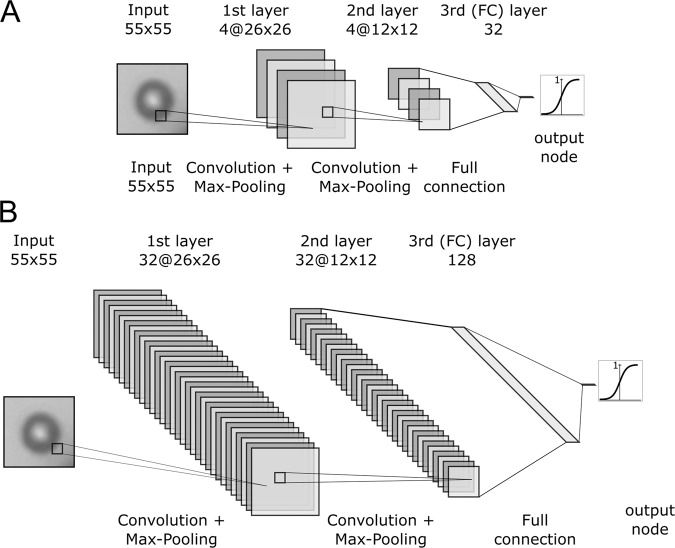
Architecture of the CNNs that were designed for classification of the images obtained from single-cell dispensing. CNN-4/32 (top) with four filters in each convolutional layer and 32 nodes in the fully connected layer, and CNN-32/128 (bottom) with 32 filters in each convolutional layer and 128 nodes in the fully connected layer. Each convolution is followed by a max pooling operation.

DeepYeast, a previously published CNN for classification of single-cell images from high-throughput microscopy^8^, was trained from scratch and validated on images obtained from the single-cell printer (scp). In order to use the model for the purpose of this work, the input shape of the first layer was set to 55 × 55 × 1 and the number of classes was reduced to two. However, the model did not perform well on the scp images. Although the training loss decreases steadily, the validation loss fluctuates strongly and increases after 100 epochs which suggests overfitting. Therefore, CNNs with a shallower architecture were applied. It turned out that models with only two convolutional layers and a single fully connected layer could be trained more robustly with regularization by random data augmentation (see Supplementary Data for details). More complex architectures did not further improve the classification performance (see Supplementary Data). This is likely due to the comparatively low complexity of the images of suspended cells obtained by the single-cell printer used in this work as compared to those images obtained from regular microscopy in microwells. In the following, a network with 4 filters in each layer and 32 fully connected nodes (CNN-4/32) and a slightly larger model with 32 filters in each convolutional layer and 128 fully connected nodes (CNN-32/128) were used as depicted in Fig. 2.

**Figure 3:**
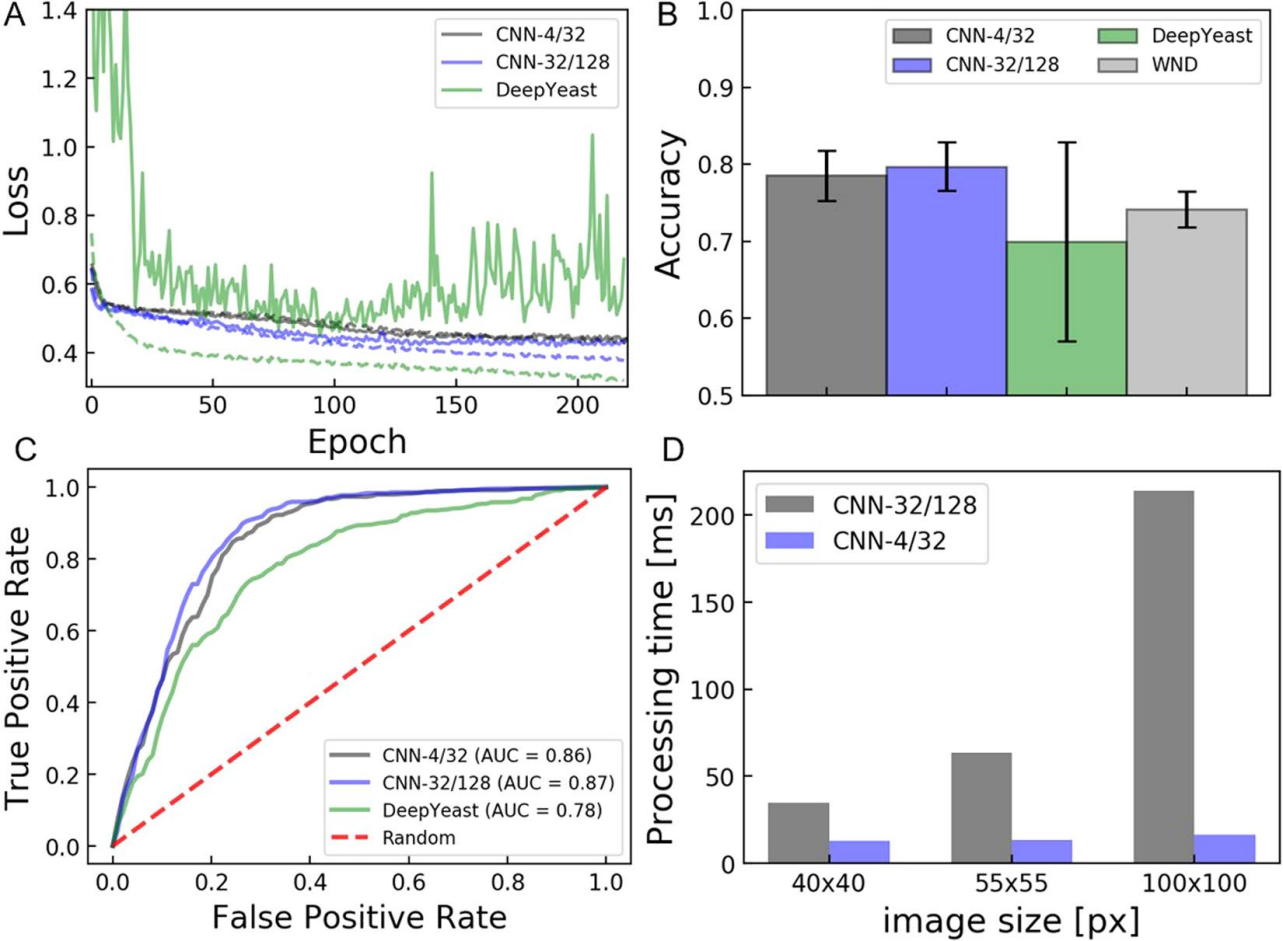
Training and classification performance. Models were trained on the CHO18all dataset with images of 55 × 55 px^2^ in size. **(A)** Training (–) and validation loss (−) over 220 epochs. **(B)** Classification accuracies of four different CNNs and the feature-based classifier WND-CHARM. **(C)** Receiver operating characteristic (ROC) curve of the CNNs. **(D)** Processing time of CNN-4/32 and CNN-32/128 for classification of a single image with different sizes. Images were processed on the CPU of a c.sight (cytena, Germany) device under full operation, i.e. while cells were dispensed.

For performance characterization the models were trained on the CHO18all dataset which contains 1423 images of CHO-K1 cells isolated with a single-cell printer. For a more detailed description of the training parameters see materials & methods and the Supplementary Data. As depicted in Fig. 3A, both the loss of CNN-4/32 and CNN-32/128 converged well during training and overfitting was strongly reduced compared to DeepYeast. Classification accuracies of 78.6% and 79.7% were archived with CNN-4/32 and CNN-32/128, respectively. This is significantly higher than the accuracies archived with the more complex network (Fig. 3B). This is likely to be caused by the low complexity of the images used for training compared to the higher complexity and higher number of the images DeepYeast was designed for. The outperformance can be also seen in the receiver operator characteristic depicted in Fig. 3C. The two shallow CNNs also outperformed the feature-based classification algorithm WND-CHARM which has been applied successfully for classification on various cell images^12^.

Since the images for cell classification to be processed in ‘real-time’ on the single-cell printer, the processing time was determined for different image sizes ranging from 40 × 40 px^2^ to 100 × 100 px^2^ **(**Fig. 3D**)**. Processing time for classification of a single image on the PC integrated into the c.sight instrument ranged from 34.8 ms to 214 ms on CNN-4/32 and from 13.2 ms to 16.5 ms on CNN-32/128. An image section of 55 × 55 px^2^ turned out to be sufficiently large to capture the single-cells while the accuracy did not significantly increase for larger sections (data not shown). Although CNN-32/128 performed slightly better, prediction on CNN-4/32 was more than four times faster (13.7 ms versus 63.5 ms). The system was operated at a dispensing frequency of 20 Hz, i.e. every 50 ms a droplet is generated. In order to keep the throughput of the instrument high, the smaller model (CNN-4/32) was used from now on, since its performance is almost as good as for the larger model.

### Additional CNN-based cell classification predicts more viable colonies and a higher throughput

Next, the CNN-based classifier for viability prediction was evaluated on four different datasets obtained from single-cell cloning experiments as summarized in Table 1. CHO18mix contained a high fraction of artificially damaged cells (see methods for details) to model a sample with low initial clone recovery. In contrast, CHO18fresh contains mainly viable cells which is also reflected by the cell images that were stored during single-cell isolation (Fig. S4). The CHO15 and CHO17 datasets were obtained from two industrial laboratories performing CLD with different single-cell printer instruments.

**Table 1:** Overview of the image datasets of CHO cells used in this work for training the models.

Dataset for Training and Validation	Number of images	Clone recovery	Device used for isolation	Comment
CHO18mix	991	22%	c.sight	mix of fresh and ‘damaged’ CHO-K1 sample
CHO18fresh	432	65.7%	c.sight	fresh CHO-K1 sample
CHO18all	1423	35.3%	c.sight	combination of CHO18mix and CHO18fresh
CHO15	755	79.3%	scp	fresh CHO-K1 sample
CHO17	1114	53.3%	scp	CHO-K1, obtained with previous version of cartridge*

The number of images corresponds to number of single-cells that were dispensed. The clone recovery corresponds to the fraction of dispensed single cells that grew to a viable colony without the new classifier. *The CHO17 dataset was obtained with a previous version of the dispensing cartridge with a higher surface roughness. This resulted in a more irregular background in the scp images.

**Figure 4:**
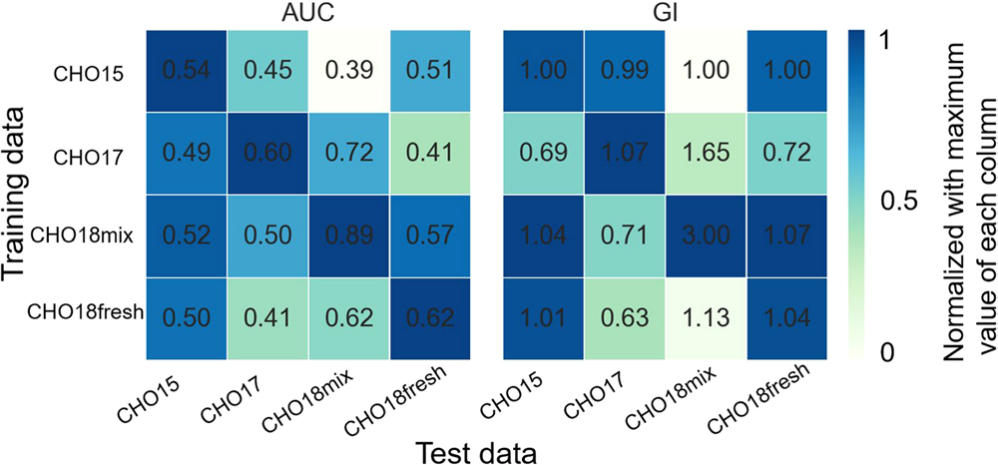
Per-column normalized heatmaps with absolute AUC and GI values. CNN-4/32 was trained on four different CHO datasets. Each of the resulting models was used for prediction of the three other datasets, respectively. The heatmaps illustrate the resulting AUC (left) and GI values (right). A GI > 1 results in more clones per plate, while a GI < 1 results in less clones per plate, if the classifier is used. For the color code the values were normalized with the maximum value of the respective column. The maximum AUC values are located on the diagonal, suggesting a specific training for each sample is necessary. For a threshold value of $T=0.5\,$and the given sample compositions, the largest growth increase for CHO18fresh and CHO15 are achieved with a classifier trained on CHO18mix.

First, the question arose whether a classifier for cell viability prediction must be trained specifically for each cell sample and single-cell printer or whether generic features exist that can be learned by the model. The latter case would allow to train a single classifier with a large dataset and apply it to different samples on different devices for viability prediction. To address this question CNN-4/32 was trained on all four different datasets. Each of the resulting four models was then used for prediction of the other three datasets, respectively. The resulting AUC and GI values are depicted as heatmaps in Fig. 4. For all samples, the maximum AUC value is located on the diagonal, suggesting that the best performance can be achieved if a classifier is specifically trained for each sample. However, the GI values show that for $T=0.5$, the largest growth increase for the CHO18fresh and CHO15 samples are obtained with a classifier trained on the CHO18mix dataset. This could be explained by the fact, that CHO18fresh and CHO15 contain mainly viable cells, while the CHO18mix contains a significant fraction of damaged cells. Therefore, a classifier trained on the CHO18mix learns more features that are related to dead cells. Interestingly, the model trained on CHO17 performed worst on the other datasets, and the other three models did not perform well in prediction the CHO17 data. This is likely due to the older version of the dispensing cartridge used for dispensing the CHO17 sample. As a result, the background is more irregular compared to the images of the training datasets. For all samples a GI > 1 could be obtained, i.e., the clone recoveries of all samples could be increased by with the additional classifier. By far the largest increase would be obtained with the CHO18mix sample that contains a large fraction of damaged cells.

**Figure 5:**
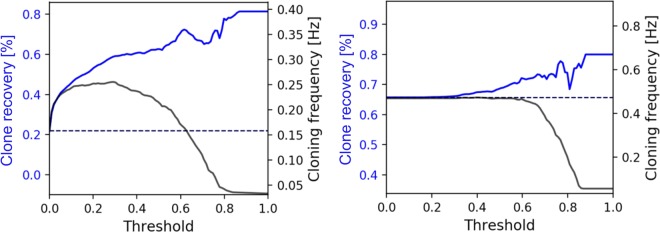
Predicted clone recovery and predicted cloning frequency as function of the threshold value. For the CHO18mix sample (left) both the clone recovery and the cloning frequency - the number of ‘viable’ cells dispensed per second - could be significantly increased with the classifier for viability prediction. The CHO18fresh (right) sample contained mainly viable cells: The clone recovery can be increased, but the process would not benefit from a higher cloning frequency.

The single output node of the CNNs used here has a sigmoid activation function (**see** Fig. 2) which outputs a floating-point value between 0 (dead) and 1 (viable) for each individual prediction. For the performance characterization above, a threshold of $T=0.5$ was applied, but the threshold is a parameter that can be set by the operator prior to cell dispensing. Intuitively, for a higher threshold value more viable clones should be selected by the classifier. However, this should also result in more viable clones that are discarded. Therefore, the predicted *clone recovery* and the predicted *cloning frequency* – the number of ‘viable’ cells that are dispensed per second - were assessed as function of the threshold value $T$. Figure 5 depicts the predicted clone recovery and cloning frequency as function of $T$ based on a model that considers the dispensing frequency of the instrument, a typical cell concentration ($1\cdot {10}^{6}$cells/ml), and processing times for cell deposition (see Supplementary Data for a detailed description). For the CHO18mix sample the clone recovery can be increased from 22% to almost 80% (GI $\sim \,$ 3.6). However, the highest cloning frequency of 0.26 Hz (+66%) is obtained at a threshold of $T\, \sim \,0.3$ which results in a GI of ~ 3. As already stated, here the process would benefit significantly from the classifier. For the CHO18fresh a clone recovery of ~75% (GI ~ 1.14) seems feasible, but for higher threshold values the cloning frequency drops quickly. The maximum cloning frequency obtained with classifier is 0.47 Hz, which is slightly lower than what would be achieved without the classifier.

### Real-time cell classification increases CHO-K1 clone recovery

**Figure 6:**
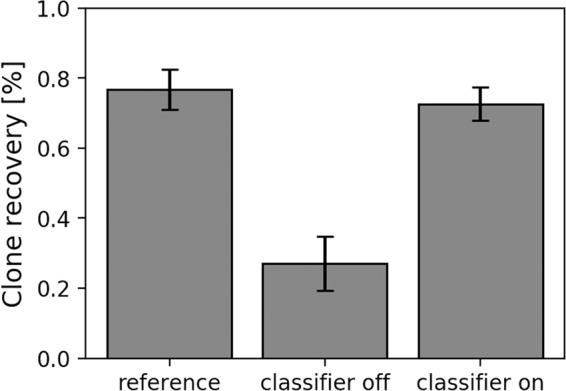
‘Real-time’ classification for viability sorting on the c.sight single-cell printer. The graph shows the clone recovery 10 days after single-cell dispensing. As a reference, single-cells from a fresh sample were dispensed without additional classifier (two 96 well plates). Next, a mixed sample containing fresh cells and damaged cells was dispensed with and without the classifier for viability sorting (three 96 well plates, each). For the classifier CNN-4/32 was trained on the CHO18all dataset and deployed on the single-cell printer. Error bars indicate the standard deviation. Threshold value was set to 0.5.

Finally, and based on the findings described above a CNN-4/32 was trained with the CHO18all dataset for 350 epochs. This model was deployed on the c.sight for ‘real-time’ image classification during single-cell printing a mixture of fresh (97% viability based on Trypan blue cell counting) and damaged CHO-K1 cells (<1% viability based on Trypan blue). As depicted in Fig. 6 the clone recovery could be increased from 27% to 73% (GI = 2.7) using the trained classifier ($T=0.5$) for selection of viable cells. This is close to the clone recovery of 76.8% achieved with freshly harvested sample that was processed as a reference. If the classifier would have been used for the freshly harvested sample, a clone recovery of 79.2% (GI = 1.03) would have been achieved. This demonstrates that machine learning based sorting can lead to significantly more viable clones for samples that contain a high fraction of dead cells, while for a sample containing mainly viable cells, only a minor increase can be obtained.

## Conclusion

As described above, drop-on-demand dispensing was combined for the first time with machine-learning based image classification for droplet sorting. The work demonstrates, that the cell images generated on a single-cell printer can be used for training of a CNN for ‘real-time’ cell classification during single-cell dispensing.

General purpose models for image classification did not perform well on the single-cell image datasets generated by the single-cell printer. A shallow network, with two convolutional layers, trained with randomly augmented minibatches, performed more robustly during training and resulted in better classification performance. This is likely due to the low complexity of the images generated in this process. An advantage of such an extremely shallow network is the low computational effort for prediction, which is important for this ‘real-time’ application. On the smaller network designed for this work, prediction on a 55 × 55 px^2^ cell image took 13.7 ms on the instrument´s CPU, which is negligible at the dispensing frequency of 20 Hz used here.

Based on the data analyzed here, it is not clear yet, whether it is feasible to train a generalized classifier that performs well on different cell samples and on different instruments. On the one hand it was found that training with a dataset that contains a significant portion of damaged cells could be beneficial – even for other samples with mainly viable cells. On the other hand, slight variations in the dispensing cartridge or optical detection system of various instruments can limit the adoption of a generalized classifier.

One classifier was trained on a mixture of freshly harvested and damaged CHO cells which was prepared as a model system. It was shown that with a threshold value of 0.5 the actual clone recovery of 22% could be increased to 66%, a 3-fold increase (GI = 3). The cloning frequency, the frequency at which viable cells are dispensed, would increase by up to 61% if the operator sets a threshold value of 0.3.

Finally, a trained classifier was deployed on a single-cell printer and applied for ‘real-time’ cell classification and sorting of a CHO-K1 sample with a large fraction of dead cells. Using the trained classifier developed in this work, the clone recovery of the sample could be increased from 27% to 73% (GI = 2.7), which is almost as high as the clone recovery of a fresh sample (76.8%). This demonstrates that the proposed implementation of CNN-based cell classification allows indeed for cell viability sorting to increase the cloning efficiency in a cell line development process.

To assess the performance of the classifiers presented here, it is important to appreciate the fact that in a typical CLD situation most of the cells are actually viable prior single-cell cloning. However, this does not mean that they will grow after single-cell isolation: Cells could be in a resting phase of the cell cycle (G0) or in another non-division state such as cellular senescence, or in an early phase of apoptosis. In all these states, a differentiation of the cells according to pure morphological features is difficult, because the cells still would appear as ‘viable’ but would not proliferate.

The additional classifier could be a great tool for the isolation of ‘difficult’ cell samples with a large fraction of stressed or damaged cells. This can be the case after cell transfection or transduction for cell line engineering, or in cases where cells must be isolated immediately after thawing which is often required for clinical samples stored in biobanks. An advantage of the low-resolution images and the usage of shallow model are the low computing time needed for training and classification. This will allow for training of a classifier on a specific sample as part of a workflow optimization in cell line development (CLD). By increasing the number of output nodes, the proposed framework can be straightforwardly adapted for multi-class classification. This could be helpful for e.g. sorting cell types.

## Materials & Methods

### Cell culture and sample preparation

Chinese hamster ovary (CHO) the cells were cultured in DME F12 medium with 10% FBS and 1% penicillin/streptomycin in a humidified 5% CO^2^ atmosphere at 37 °C. Upon confluence cells were detached with trypsin and washed 2 times in phosphate buffered saline (PBS). For isolation of highly viable cells single-cell printing was performed within 3 hours while the cells were kept on ice. Prior loading 40 µl of the sample into the cartridge of the single-cell printer the concentration was adjusted to 10^6^ cells/ml by dilution with PBS. To model cell damage due to sample preparation, after resuspension in PBS cells were vortexed for 30 seconds at the highest setting (Vortex-Genie 2, Scientific Industries) and subsequently stored at room temperature for four days. Prior cell isolation the sample was washed two times with PBS, adjusted to 10^6^ cells/ml and mixed 1:1 with a freshly harvested CHO sample if not stated otherwise.

### Single-cell printing

Single-cell isolation was performed on a c.sight single-cell printer (cytena, Germany) without additional hardware modifications. Some experiments were performed on a scp (cytena, Germany) and on a former prototype^6^ to investigate the impact of hardware variations. To retrieve the cell images for training the x.sight software was modified: For each dispensed single cell a region of 300 × 300 or 100 × 100 pixels around the respective cell was cropped and written to the hard drive together with the well ID. For a posteriori background removal, the 2 images before the ejection event were stored as well.

### Clonal expansion and assessment of colony growth

Cells were dispensed into 96-well plates prefilled with 180 µl growth medium. After single-cell deposition the plates were incubated for 10 days in a humidified 5% CO^2^ atmosphere at 37 °C. The growth of viable colonies was assessed manually using an inverted microscope with 4x objective. Cells that resulted in visible colonies were regarded as ‘viable’. The clone recovery is defined as number of viable colonies divided by the number of wells populated with single cells (96 per plate).

### Datasets and image preprocessing

Several image datasets were used in this study as summarized in Table 1. Two datasets were collected by isolation of single CHO cells with a c.sight device. The other two image datasets were obtained previously on a scp single-cell printer. For training of the CNNs, the cell images from the single-cell dispensing experiments were further cropped to 55 × 55. The background was removed by subtracting the ‘empty’ images captured just before the respective cell was imaged in the nozzle and dispensed from the cell image.

### Feature-based image classification with WND-CHARM

Classification performance of the CNNs were compared to WND-CHARM (version 1.60) which is a multi-purpose feature-based classification algorithm^12^. First, the algorithm computes 1025 features from the images with:

*wndchrm train*

Second, classification metrics are computed using unbalanced training and 10-fold cross-validation with:

*wndchrm test -r#0.9 -n10*

### Implementation and training of the CNN models

Python scripts were written to extract, label, and sort the image data. CNNs were implemented and trained using the Python deep learning library Keras (https://keras.io) which is run on top of Tensorflow^13^. Training was performed on a NVIDIA GeForce GTX 960 GPU using a mini-batch approach. This means, for training one epoch on a training set of N images, i batches of n = batch size images are passed through the network in i iterations such that i = N/n. In total a model was trained for $I=e\cdot \dot{i}$ iterations, where e is the number of training epochs. Since the batch size has a significant effect on the generalization performance and convergence of the model^14^ it was treated as hyper parameter that was to be fine-tuned. Class weighted binary cross-entropy was used for the loss function. scikit-learn^15^ was used to calculate classification performance metrics and for splitting the data into training and validation sets. Each combination of model and dataset was investigated by 10-fold cross-validation. That means the dataset is split into k = 10 subsets and training is perform k-fold on a training set comprising k-1 subsets while 1 subset is hold back for validation. Classification performance metrics (accuracy, AUC, etc.) of the models were then calculated as mean value of the k folds. Results were visualized with the python libraries Pandas and matplotlib. For ‘real-time’ classification during single-cell printing, trained models were exported into the protobuf format. The ‘frozen’ models were then imported into a modified version of the c.sight software using tensorflowsharp, a TensorFlow API for.NET languages.

## Supplementary information

Supplementary Information.
