# Supplementary material for: Single-cell dispensing and ‘real-time’ cell classification using convolutional neural networks for higher efficiency in single-cell cloning
Source: Sci Rep. 2020 Jan 27;10:1193. doi: 10.1038/s41598-020-57900-3 (PMC6985245; doi:10.1038/s41598-020-57900-3)
Supplement: Supplementary file 1 — Supplementary Information. [file 41598_2020_57900_MOESM1_ESM.docx]

**Supplementary data**

**Single-cell dispensing and ‘real-time’ cell classification using convolutional neural networks for higher efficiency in single-cell cloning**

Julian Riba, Jonas Schoendube, Stefan Zimmermann, Peter Koltay, Roland Zengerle

**Network architecture**

After training and validation of MobileNetv2 (data not shown here)^1^ and DeepYeast^2^ it became obvious, that the low-resolution images obtained from the single-cell printer require a shallower architecture to avoid overfitting. Therefore, four architectural parameters of the model were investigated systematically: The number of convolutional layers, the number of filters used in each convolutional layer, the number of fully connected layers, and the number of nodes in the fully connected layer. The results are summarized in **Figure S1**. Overall, in can be observed, that the classification accuracy can decrease for more than 2 convolutional layers (**Figure S1 A**). The number of fully connected layers have only a very modest impact on the performance (**Figure S1 B**). A drop in classification accuracy was observed for a model with 16 dense nodes in the fully connected (FC) layer compared to 32 or 128 nodes (**Figure S1 C**). Further, increasing the number of filters from 4 to 8, 16, or 32 did not have much influence on the classification accuracy (**Figure S1 D**). For ‘real-time’ image classification on the single-cell printer a smaller network is preferable, as lager networks result in longer processing times, which decrease the throughput of the instrument. Therefore, a shallow model with 4 filters, 32 dense nodes (CNN-4/32) was selected. In order to assess how the network size influences the processing times a second slightly larger model with 32 filters and 128 dense nodes (CNN-32/128) was also selected for further characterization. I should be noted here, that the low-resolution images of 55x55 px^2^ that were used here, restrict the number of pooling operations that can be used in a senseful manner, which is one explanation why MobileNetv2 and DeepYeast did not perform well. This could be addressed by upsampling the images prior training and prediction. Here, we did not opt for this strategy, since this would result in significantly longer CPU processing times.


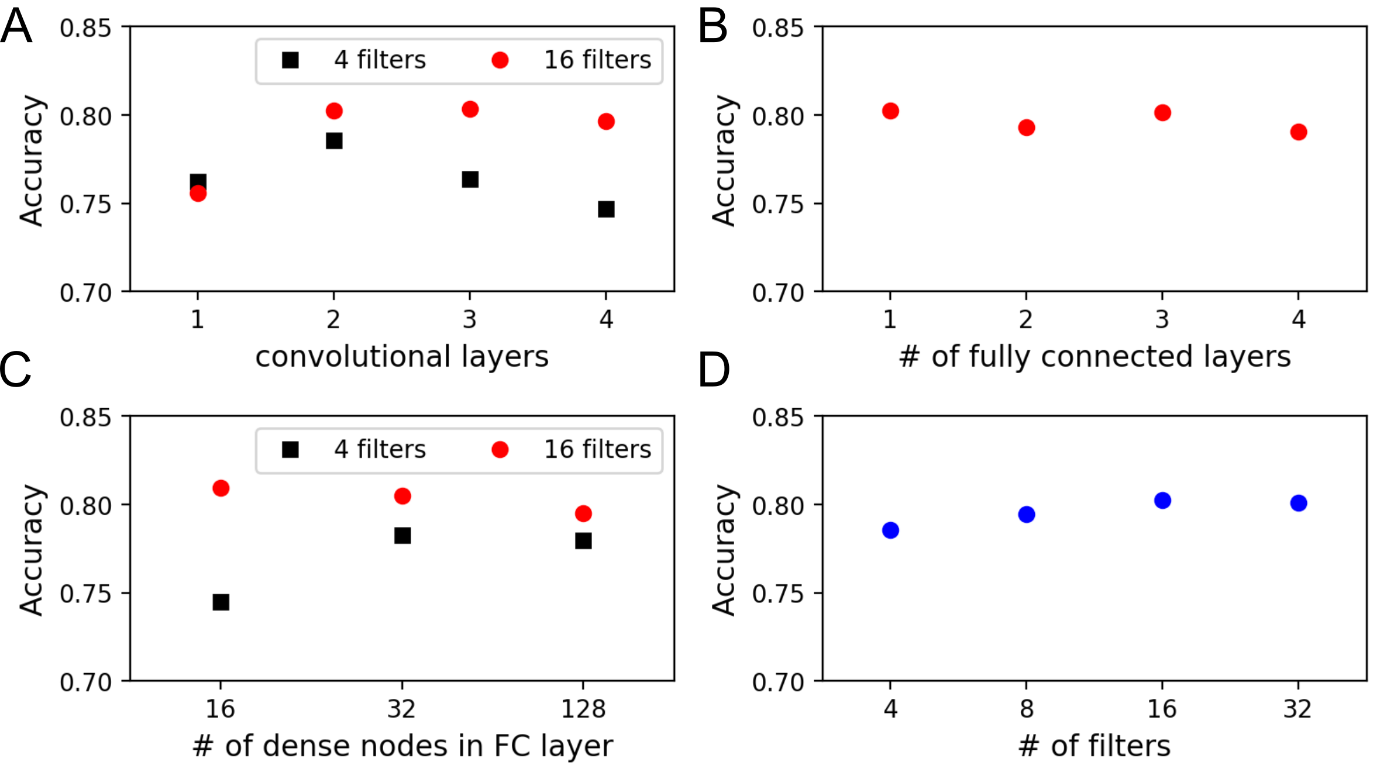


**Figure S1:** Influence of the network architecture on the validation accuracy. The model architecture was varied systematically. Training and validation were performed on the CHO18all dataset. If not otherwise stated in the plots, the models shown here have 2 convolutional layers which 16 filters each, and 2 fully connected (FC) layers with 32 nodes. **(A)** Two convolutional layers are required but more layers can result in lower classification performance, even for a very narrow model with only 4 filters. **(B)** A single fully connected layer is enough. **(C)** A drop in classification accuracy was observed for a model with 16 dense nodes in the fully connected (FC) layer. **(D)** The number of filters has only a small influence on the classification accuracy. Here, the performance doesn´t increase for more than 16 filters. Note that all filters have a kernel with a size of 3x3.

**Data augmentation**

A common method to reduce overfitting on image data is to artificially enlarge the training dataset by applying geometric transformation to the original images^3^. Here, overfitting could be significantly reduced (**Figure S2 A**) by moderate geometric data augmentation. For each minibatch the original cell images were subject to a random vertical flip and to a translation in the vertical and horizontal direction by a random distance between 0 and 5 pixels as shown in **Figure S2 B**. Such a moderate translation ensures that the model can robustly classify images in which the cell is not perfectly centered. On the other hand, it makes sure that all translated cells are completely captured by the image. To rule out any influence of the nozzle geometry in the background no rotations were applied to the images. However, as the nozzle geometry is vertically symmetric, vertical flip seemed reasonable. The other two affine transformations scaling and shear were not used, since the size and roundness of a cell can be relevant morphological characteristics that should be retained.


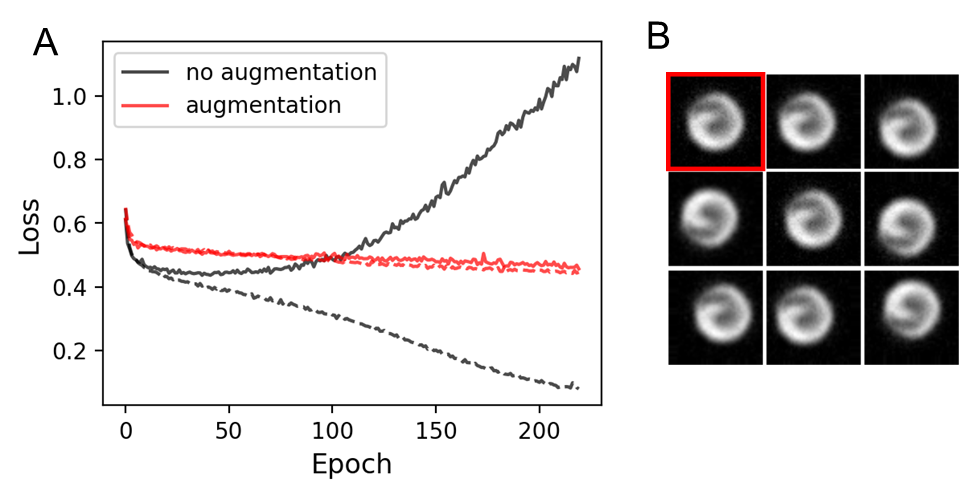


**Figure S2:** Moderate data augmentation by geometric translations that was randomly applied to the training images significantly removed overfitting as shown by the training curves of CNN-4/32 on the CHO18all dataset in (**A**): Without augmentation the validation loss (solid line) started to increase dramatically after ~50 epochs. With the random augmentation strategy, the validation loss reduces with the training accuracy (dashed). Almost no overfitting occurs. Eight such randomly translated cell images are shown in (**B**), the original cell image is highlighted in red. The grayscale intensities of the background subtracted images was scaled up for better visibility.

**Tuning of hyperparameters for training**

For training two different optimizers were tested. It turned out that the optimizer had a significant influence on the convergence during training and on the final classification performance. Using stochastic gradient decent (SGD) the best results could be achieved with a learning rate of 0.006, and when a Nesterov momentum of 0.7 was applied. Although with these settings the models converged well during training, training with Nadam always resulted in a smaller loss and therefore a better classification performance. Nadam^4^ is a variant of SDG which makes use of a Nesterov momentum and an adaptive learning rate.


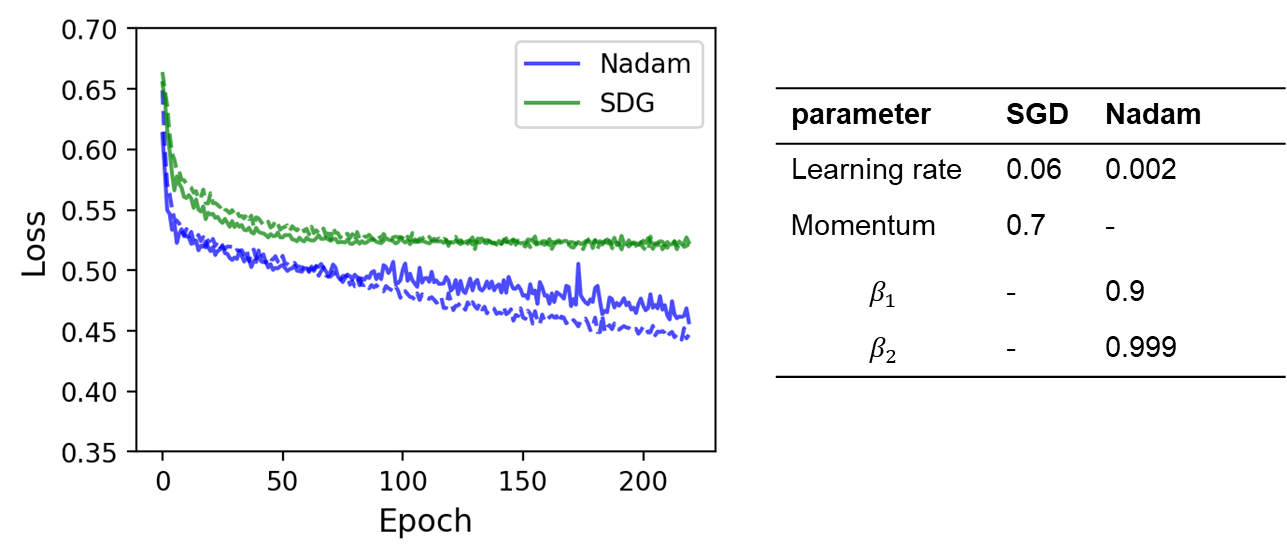


**Figure S3**: Influence of two different optimizers on the training (dashed) and validation loss (solid). Here CNN-4/32 was trained on the CHO18all dataset Although the losses converged well, they could be reduced significantly by using the adaptive optimization algorithm Nadam. The parameter used for the optimizer are summarized in the table. For Nadam they were chosen as recommended in the original publication ^4^.

**Calculation of the predicted clone recovery and cloning frequency**

Based on predictions on the validation data, a predicted clone recovery can be calculated, which is the number of viable colonies that would have been obtained if the classifier would have been used for cell viability sorting. The number of viable colonies that are predicted to grow is given as,

$$c_{v}\cdot TPR.$$

As explained before, the total number of dispensed cells yields

${c_{v} TPR}+ (1 - {c_{v})(1- FPR})$.

Therefore, the predicted clone recovery can be expressed as:

Clone recovery $= \frac{TPR \cdot c_{v}}{TPR \cdot c_{v} + FPR (1- c_{v})}$.

The frequency, at which cells able to grow to colonies are dispensed, can be derived by considering the rate at which growing cells are printed divided by the average time that is needed to dispense a single cell:

$$\text{Cloning frequency} = \frac{TPR \cdot c_{v}}{t_{sc} + t_{pred}+{(t}_{vac}+t_{axis})\cdot(TPR \cdot c_{v}+FPR \cdot{(1- c}_{v}))}$$

It must be noted, that the denominator is the sum of the average time for a single cell to appear in the nozzle of the dispenser $t_{sc}$, the time needed to run the prediction $t_{pred}$, and the time for dispensing a single cell. The latter is the sum of the time $t_{vac}$related to switching the vacuum shutter and time $t_{axis}$ for the axis movement to approach the next well must be multiplied by the fraction of cells that are selected for dispensing. The average time for a single cell to appear in the nozzle of the dispenser $t_{sc}$ is derived by multiplying the dispensing frequency $f_{d}$ with the Poisson based droplet loading:

$P_{\lambda}\left( k \right)= \frac{\lambda^{k}}{k!}e^{-\lambda}$,

where $k=0, 1, 2, 3, \ldots$ is the resulting number of cells per well, and λ the average number of cells per droplet ($\lambda= c\cdot V_{d}$). For a given cell concentration c and droplet volume $V_{d}$ the Poisson loading yields $P_{\lambda}\left( k=1 \right)= \lambda e^{-\lambda}$.

The parameters used in the model are summarized in the following table:


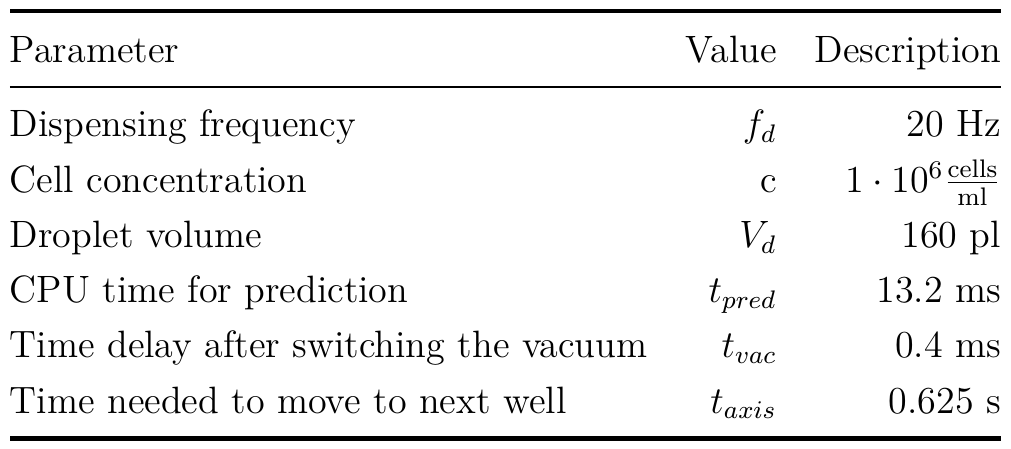


**Table S1:** Model parameter for calculation of the predicted cloning frequency, i.e. the number of viable cells the grow to clonal colonies that are dispensed per second


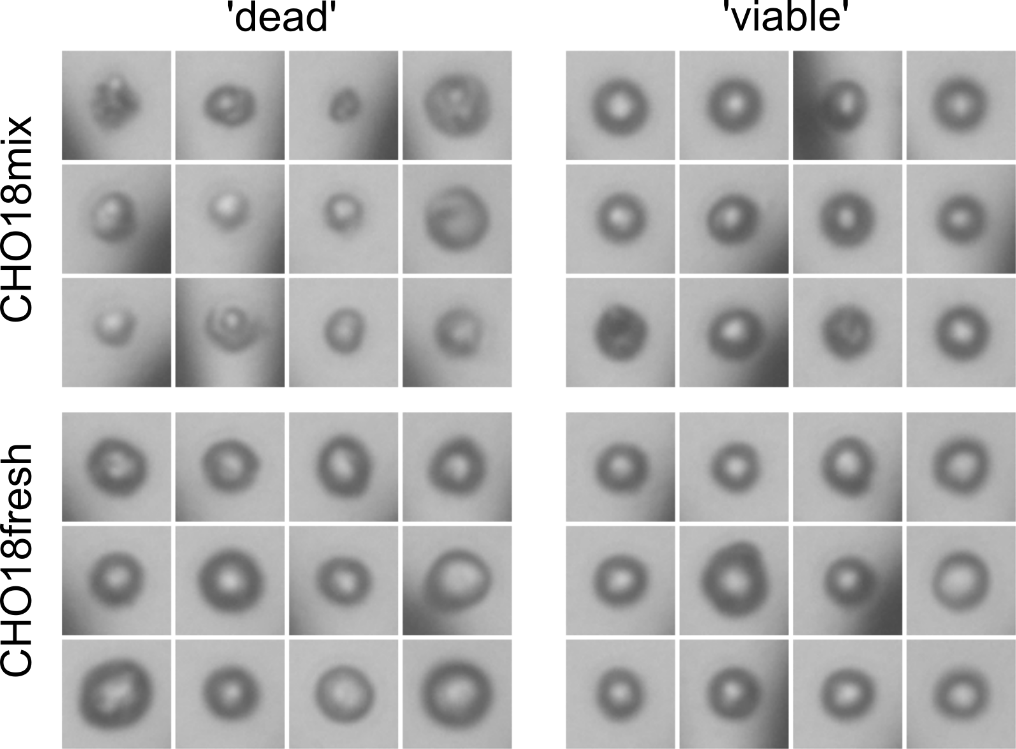


**Figure S4**: A selection of cell images from CHO18mix sample (top), and from the CHO18fresh sample (bottom). From each sample 12 ‘dead’ cells that did not result in colony growth and 12 ‘viable’ cells that resulted in colony growth were randomly selected from the datasets.

**References**

1. Sandler, M., Howard, A., Zhu, M., Zhmoginov, A. & Chen, L.-C. MobileNetV2: Inverted Residuals and Linear Bottlenecks. *Proc. IEEE Conf. Comput. Vis. Pattern Recognit.* 4510–4520 (2018). doi:10.1134/S0001434607010294

2. Pärnamaa, T. & Parts, L. Accurate Classification of Protein Subcellular Localization from High-Throughput Microscopy Images Using Deep Learning. *G3* **7**, 1385–1392 (2017).

3. Krizhevsky, A. & Hinton, G. E. ImageNet Classification with Deep Convolutional Neural Networks. *Adv. Neural Inf. Process. Syst.* 1097–1105 (2012).

4. Dozat, T. Incorporating Nesterov Momentum into Adam. *ICLR 2016 Work.* (2016). doi:10.1016/j.ahj.2004.02.019
